# Management of the Female With Non-classical Congenital Adrenal Hyperplasia (NCCAH): A Patient-Oriented Approach

**DOI:** 10.3389/fendo.2019.00366

**Published:** 2019-06-06

**Authors:** Sarantis Livadas, Christina Bothou

**Affiliations:** ^1^Metropolitan Hospital, Pireas, Greece; ^2^Department of Endocrinology, Diabetology and Clinical Nutrition, University Hospital Zurich, Zurich, Switzerland

**Keywords:** premature pubarche, PCOS, androstenedione, 17OH progesterone, CYP21A2

## Abstract

Non-classical congenital adrenal hyperplasia (NCCAH) is considered to be a common monogenic inherited disease, with an incidence range from 1:500 to 1:100 births worldwide. However, despite the high incidence, there is a low genotype-phenotype correlation, which explains why NCCAH diagnosis is usually delayed or even never carried out, since many patients remain asymptomatic or are misdiagnosed as suffering from other hyperandrogenic disorders. For affected adolescent and adult women, it is crucial to investigate any suspicion of NCCAH and determine a firm and accurate diagnosis. The Synacthen test is a prerequisite in the event of clinical suspicion, and molecular testing will establish the diagnosis. In most cases occurring under 8 years of age, the first symptom is premature pubarche. In some cases, due to advanced bone age and/or severe signs of hyperandrogenism, initiation of hydrocortisone treatment prepubertally may be considered. Our unifying theory of the hyperandrogenic signs system and its regulation by internal (hormones, enzymes, tissue sensitivity) and external (stress, insulin resistance, epigenetic, endocrine disruptors) factors is presented in an attempt to elucidate both the prominent genotype-phenotype heterogeneity of this disease and the resultant wide variation of clinical findings. Treatment should be initiated not only to address the main cause of the patient's visit but additionally to decrease abnormally elevated hormone concentrations. Goals of treatment include restoration of regular menstrual cyclicity, slowing the progression of hirsutism and acne, and improvement of fertility. Hydrocortisone supplementation, though not dexamethasone administration, could, as a general rule, be helpful, however, at minimum doses, and also for a short period of time and, most likely, not lifelong. On the other hand, in cases where severe hirsutism and/or acne are present, prescription of oral contraceptives and/or antiandrogens may be advisable. Furthermore, women with NCCAH commonly experience subfertility, therefore, there will be analysis of the appropriate approach for these patients, including during pregnancy, based mainly on genotype. Besides, we should keep in mind that since the same patient will have changing requirements through the years, the attending physician should undertake a tailor-made approach in order to cover her specific needs at different stages of life.

## Definitions and Prevalence

Congenital adrenal hyperplasia (CAH) encompasses a family of autosomal recessive disorders characterized by mild to acutely impaired cortisol synthesis due to a deficiency in one of the five adrenal steroidogenic enzymes required for cortisol production (1, 2). Conventionally, CAH is divided into (a) classical (CCAH), presenting with salt-wasting or the simple virilizing form that is manifest at birth and/or in the neonatal period, and (b) non-classical (NCCAH), representing a less severe form of the disorder which lacks genital ambiguity, is not immediately life-threatening, and presents later in life, or remains asymptomatic, or is misdiagnosed as a different disease (3). However, the boundaries across the different forms of CAH are not strictly defined, which tends to increase the challenges associated with this disorder. It is therefore advisable to consider CAH as a continuum of phenotypes, from severe to mild or else asymptomatic (3).

The most common form of CAH is caused by 21-hydroxylase deficiency (21OHD), resulting in impaired or no conversion of 17 hydroxyprogesterone (17 OHP) to 11 deoxycortisol and of progesterone to deoxycorticosterone. The blockade of steroid conversion results in increased production of androgen precursors, under CRH-ACTH stimulation, leading to biochemical hyperandrogenism, marked by elevated 17-OHP levels. The classical form of the disease, occurs in 1 out of 16,000 live births worldwide. However, it should be mentioned that the perceived incidence of the disease is related to the screening method used. For example, it has been reported that the incidence of the classical form of CAH in Sweden is 1:11,500 when a case survey approach is used, which figure however drops to 1:9,800 when hormonal screening is applied. Likewise, the incidence in France and Switzerland ranges between 1:15,472 and 1:23,000 and 1:10,749 and 1:11,661 when the different methods of screening are used (4).

NCCAH is much more frequent, occurring in approximately 1 out of 1,000 Caucasians and more commonly in certain ethnic groups, such as Ashkenazi Jews (1:27), Hispanics (1:53), Yugoslavs (1:62), and Italians (1:300) (5, 6). Nevertheless, data on the prevalence of NCCAH based on different estimation methods (case-survey, hormonal screening, and molecular testing) are lacking. NCCAH is considered the most common autosomal recessive endocrine disorder with a carrier frequency of 1:25 to 1:10. In NCCAH due to 21-OHD, the residual enzymatic activity is estimated to be about 10–70% (7–9).

## Diagnosis

In comparison to the diagnosis of the classical form of the disease, which is made at birth or during the neonatal period because of genital ambiguity and/or salt-wasting symptoms or through screening programs employed in some countries most cases of NCCAH are not easily detectable (4, 10). Additionally, many individuals remain asymptomatic during childhood and adolescence, have normal reproductive function, and only become aware of NCCAH due to the diagnosis of another family member and consequent testing (11). However, most women with NCCAH seek medical assistance when they experience symptoms of androgen excess and, when clinical suspicion prompts testing, elevated basal 17 OHP levels will more likely than not point to a diagnosis of NCCAH.

Indeed, the clinical guidelines proposed by the Endocrine Society recommend a baseline non-stimulated value of 17 OHP as the screening tool for NCCAH. Morning 17 OHP levels >6 nmol/L in the follicular phase in menstruating females should prompt further evaluation, since it has been shown that levels above 6 nmol/L capture 90% of NCCAH individuals (12, 13). Random measurements of 17 OHP have not been shown to be helpful, since these often yield normal levels in patients with NCCAH, and they are, moreover, extremely high in the luteal phase in half of healthy females (13). However, in our data derived from 280 subjects with the disease, six patients (2.1%) had a baseline 17 OHP value < 6 nmol/L (14). Additionally, Bidet et al., in a large cohort of women with NCCAH verified by molecular techniques also found basal 17 OHP values lower than 6 nmol/L in 8% of the subjects studied (15). Finally, based on data collected by Speiser et al., 9% of individuals with NCCAH displayed 17 OHP values lower than 2 ng/ml (that corresponds to 6nmol/L). According to other studies, a baseline value of 17 OHP between 5.1 and 9 nmol/L is sufficient for the diagnosis of NCCAH (13, 16, 17). Recently, a level of basal 17 OHP of 4.6 nmol/L was suggested as a threshold for ACTH testing to predict NCCAH in subjects with premature adrenarche during childhood (18).

The sum total of these findings and suggestions indicates that the selection of patients who will undergo a Synacthen stimulation test should be evaluated case by case. A 17 OHP post-stimulation level over 3 nmol/L is required for the diagnosis (19). Heterozygotes carrying one CYP21A2 mutation exhibit slightly elevated 17 OHP levels post ACTH stimulation, though there is overlap in unaffected subjects (9). However, as Dacou-Voutetakis et al. have pointed out, if the sum of basal and post-stimulation 17 OHP values exceeds 1.5 nmol/L, then the possibility of heterozygosity is exceptionally high (20).

Another important consideration regards the techniques used for 17 OHP evaluation. 17 OHP levels are measured by a variety of immunoassay methods, but as has recently been shown, the most accurate and reliable results were achieved by the implementation of the combination of liquid chromatography with mass spectrometry (LC-MS/MS). Indeed, many false positive 17 OHP measurements were found when LC-MS/Ms measurements were compared with standard methods (21) Nevertheless, the latter procedures are not universally used, in the event of an uncertain diagnosis, they will provide more precise results. Of note, the screening and diagnostic thresholds for LC-MS/MS quantified 17 OHP are yet to be defined. These thresholds are likely to be lower than those established with immunoassays due to the enhanced specificity of LC-MS/MS, a method less prone to cross-reactivity and interferences (22, 23). Whether a urinary steroid profile is required for the definitive diagnosis remains to be elucidated (22). In borderline cases, it is advisable to obtain a complete adrenocortical profile after the ACTH stimulation test to differentiate 21-hydroxylase deficiency from other enzyme defects and establish a firm diagnosis.

Specifically, a complete steroid profile should be performed in equivocal cases to confirm 21-hydroxylase deficiency and exclude other enzymatic detects. Inclusion of 17 OHP, cortisol, 11-deoxycorticosterone,11-deoxycortisol,17-OH-pregnenolone, Dehydroepiandrosterone, and androstenedione in a serum steroid profile would be useful to exclude other causes of CAH such as 11β-hydroxylase deficiency and, more rarely, 3β-hydroxysteroid dehydrogenase deficiency or P450 oxidoreductase deficiency (24). While genetic testing is not considered to be a primary diagnostic tool for NCCAH at this time, it is mandatory for diagnosis confirmation and for genetic counseling (3).

As far as the cortisol levels are concerned, generally a post-stimulation value of atleast 496 nmol/L is expected. Of note, according to Stoupa et al., 60% of 47 children with NCCAH as a result of 21 OHD had low cortisol values after the stimulation, a finding pointing to the need for increased surveillance for the development of adrenal insufficiency during major stressor events (25).

## NCCAH Manifestations in Women

The clinical expression of NCCAH is characterized by a high level of polymorphism as concerns not only age of onset but also the different signs and symptoms. It is reported that the first clinical presentation of NCCAH is in 11% of cases before the age of 10 years and in 80% between the ages of 10 and 40 years (12). The genotype-phenotype correlation in CAH and NCCAH has not as yet been elucidated. Speiser et al. suggest that most but not all of the phenotypic variability in 21-hydroxylase deficiency results from allelic variation in CYP21A2 (26).

### Manifestations in Childhood

In the newborn period, typically NCCAH females remain asymptomatic and have normal external genitalia. The earliest case of NCCAH reported is a 6 month old girl who developed pubic hair (11). Usually, clinical findings and symptoms in NCCAH cases start from the age of 5 years or even later and are related to increased androgen levels, though mild cortisol deficiency can also occur in some cases (8). In 92% of NCCAH cases, the first symptom that manifests is premature pubarche (PP), which occurs under 8 years of age in girls and under 9 years in boys (12) with an estimated prevalence of between 10 and 11.3% (15). Of note, in children referred with PP, according to different studies, the incidence of NCCAH ranges from 5 to 30% (20, 27). However, based on our analysis of 280 patients with molecular confirmation of NCCAH, we found that the incidence of PP in 94 females younger than 8 years was as much as 88%. On the other hand, an analysis of 45 males with NCCAH identified PP in only 29% of subjects (14). Furthermore, we should keep in mind that about half of subjects with PP may be heterozygote carriers of a CYP21A2 mutation.

Another aspect that needs clarification is the relationship between PP and precocious puberty. It is hypothesized that in some cases, the peripheral aromatization of adrenal androgens to estrogens may activate the hypothalamic-pituitary-gonadal axis, leading to secondary precocious puberty (28). In such an instance regular follow-up is needed. Some children develop hirsutism, acne, and/or rapid growth and manifest tall structure and bone age advancement (29). The latter can result in truncated final height as a consequence of rapid epiphyseal fusion. Other rare clinical findings during childhood include labial adhesion, perianal hair, clitoromegaly, and hoarseness of voice (30–32).

### Manifestations in Adolescence and Adult Life

During adolescence and adulthood, NCCAH in women presents with hyperandrogenic symptoms resembling polycystic ovary syndrome (PCOS) (33). Symptoms include hirsutism, acne, androgenic alopecia, anovulation, menstrual dysfunction, and infertility. In a multicenter study, the most common symptoms among adolescent and adult women were hirsutism (59%), oligomenorrhea (54%), and acne (33%). Among 161 women with NCCAH, presenting symptoms were hirsutism (78%), menstrual dysfunction (54.7%), and decreased fertility (12%) (34). In our group of 161 women with the disease 63% of the patient presented with a polycystic ovary-like phenotype (14). This finding has a *vice versa* implication, since the incidence of NCCAH is high in women with hyperandrogenaemia. Certainly, the study by Witcel et al. reporting an incidence of NCCAH ranging from 1 to 33% in hyperandrogenic women is of great interest. In another study of 950 women who were referred for evaluation of androgen excess, NCCAH was detected in 4.2% of them. However, the NCCAH patients were younger and more hirsute compared with the other groups of hyperandrogenic patients. They were also characterized by significantly higher levels of testosterone, free testosterone, and 17 OHP than patients with other hyperandrogenic syndromes. In ovarian ultrasound, 77% of them displayed polycystic ovaries and 41% increased ovarian size. In sum, they fulfilled the PCOS criteria according to NIH and Rotterdam at a percentage of 56 and 72.8%, respectively, (35).

The progressive nature of the disease is highlighted by the fact that the prevalence of hirsutism has been shown to increase with age and has been observed to be rare before puberty. Hirsutism is the most common clinical manifestation reported in patients with NCCAH, ranging from 71 to 96% (28, 36). Pubertal girls with NCCAH typically present with hirsutism (37, 38). At the other end of the spectrum is the issue of alopecia. Male pattern balding is reported in NCCAH patients. The prevalence of alopecia also appeared to increase with age, from 6% in patients during the second decade of their life to 19% in the fifth, indicating again the progressive nature of the disease (39). Acne is reported in almost 33% of NCCAH subjects (36). Remarkably, mutations of the CYP21A2 gene causing NCCAH were detected in 4.9% of 123 adult females presenting with severe acne (40).

Menstrual irregularities including oligomenorrhea or secondary amenorrhea can often be the presenting sign of NCCAH in post menarche individuals. Additionally, 8–9% of cases also experience primary amenorrhea and, for this reason, seek medical advice for the first time. Moreover, among NCCAH individuals, oligomenorrhea is more common than primary amenorrhea. For example, in a series from Moran et al., among NCCAH women aged 10 to 19 years, oligomenorrhea was present in 56% of the cases in comparison to only 9% of adolescence with primary amenorrhea (12). To conclude, so many NCCAH symptoms resemble those of PCOS that it is not surprising that NCCAH has been dubbed the big mimicker of PCOS. In any case, a diagnosis of NCCAH should be considered during the evaluation of any young woman who is referred for hyperandrogenic symptoms.

In a study by Arlt et al., patients with NCCAH were characterized by significantly higher BMI and lower final height in comparison to matched controls (41). These data, in conjunction with a few studies that indicate the presence of insulin insensitivity, suggest an unfavorable metabolic profile that could be pathophysiologically explained by the effect of elevated androgen concentrations and/or as a result of glucocorticoid therapy (42, 43). Osteoporosis can also be detected in these subjects, probably as a consequence of the corticosteroid therapy (34).

## NCCAH Management From the First Manifestation to the Adult Life

Once the diagnosis of NCCAH has been established, several issues related to subsequent treatment recommendations warrant consideration. The first question to be addressed is whether glucocorticoid (GC) therapy is indicated. The general reasoning behind this approach is that by providing sufficient cortisol concentrations to the patient, her daily needs are covered and, consequently, CRH-ACTH axis stimulation will be tapered, leading to decreased adrenal androgen production. The general rule concerning this approach is that GCs are given at replacement and not pharmacological doses, while the influence of age, gender, laboratory data, patient-specific recommendations, and goals of treatment on glucocorticoid replacement therapy are seriously taken into consideration. However, we should also be aware that prolonged GC substitution therapy may lead to hypercortisolemia, with the resultant well-documented adverse effects on every aspect of metabolism, especially growth, fat distribution, and insulin resistance as well as on psychological profile. Another major disadvantage of this approach is the lack of an adequate clinical index or biochemical marker of adequate replacement dosage, such as exist regarding TSH values in hypothyroidism.

Finally, the fact that there is no consensus as to which GC should be used and the absence of long-term data regarding different modalities of GC administration further complicate attending physicians' decisions on and selection of the optimal choice for each patient. Hydrocortisone is typically used in children, as it most closely resembles the natural hormone (cortisol), but it is not considered a suitable approach in adolescents and young females due to the need for multiple daily dosing. Hence, most adult endocrinologists prefer either dexamethasone or prednisolone, at appropriate doses. On the other hand, it must be pointed out that the equivalence of different GCs is based on their anti-inflammatory action and not on different aspects of human metabolism. Since cortisol affects almost 20% of the human genome, diverse responses of different GCs are expected in various tissues. Corroborating this perception is the fact that the administration of dexamethasone to patients with CAH has shown deterioration of indices of insulin resistance in comparison to other GCs (44). Additionally, the administration of 2.5–7.5 mg of prednisolone, a dose considered as normal, exerts a longstanding negative impact on bone metabolism (45). Therefore, hydrocortisone should be considered the best form of treatment in cases of GC supplementation therapy. The advent of the newly synthesized hydrocortisone formula with one pill per day and its initial positive results in patients with CAH shows much promise for the future (46).

### Management During Childhood

NCCAH patients who are diagnosed during childhood with signs of PP may be treated with hydrocortisone with the aim of suppressing the adrenal hormones and preventing rapid advancement of bone age that could affect final height. In those patients in whom hydrocortisone treatment was initiated 1 year before the onset of puberty and who had a bone age below 9 years, final height remained within the genetic potential (47). Available studies indicate that adult height approached the expected target height in patients that were closely monitored and who strictly complied with medication plans (48–50). A recent small study of five cases (patients 6.1–9.2 years of age) demonstrated that an ultralow dose of dexamethasone is a promising option to reduce endogenous stress and its effects. Whether this constitutes a realistic approach and how much the administered dose should be remain to be elucidated (51). A study by Nebesio et al. compared the hormonal effects and pharmacokinetics of hydrocortisone, prednisolone, and dexamethasone in 9 prepubertal patients with CCAH. They showed that dexamethasone was more potent that the other forms in achieving significantly lower adrenal hormone levels, hence suggesting that dexamethasone is more efficacious for the suppression of adrenal androgen production (46). Of course, further studies are needed to verify or reject this finding. Furthermore, in some cases, elevated androgen concentrations may lead to secondary stimulation of the GnRH axis, leading to premature puberty. In such a case, a parallel course with GnRH analog may be prescribed if bone age is significantly higher than chronological age and/or projected final height is disproportionate to target height.

Another important consideration regards cases of concomitant GH deficiency in the event that treatment with GnRH analogs and GH prescription resulted in attainment of target height (52). According to the current guidelines, the above-described therapy is cautiously recommended only for those cases in which the predicted height SD is −2.25 < the target height or even lower (53). An alternative indication for starting hydrocortisone treatment is an inadequate cortisol response post ACTH stimulation (36).

The preferred GC treatment in children is usually hydrocortisone 10–15 mg/m^2^, divided into three doses. Often, lower doses have also proved effective, starting from 6 mg/m^2^/day (34). Overdosing should be avoided, considering it that can result in poor growth as well as Cushingoid features. Regular growth pattern, a bone age compatible to chronological age, and absence of central obesity may also serve as clinical indices for appropriate management. Regarding biochemical/ hormonal profile, androstenedione and testosterone levels in the mid to upper ranges for bone age are considered better markers of adequate GC replacement therapy in children with NCCAH. However, suppressed testosterone levels were found in 10% of NCCAH patients, whereas another 28% of patients had increased testosterone concentrations, this phenomenon possibly being attributable to hydrocortisone variability (54, 55). DHEAS values are decreased with very small doses, whereas the suppression of 17 OHP and progesterone levels requires very high GC doses. It should be noted that 17 OHP values are crucial for the diagnosis, but not helpful during follow–up. Of note, blood sampling for hormonal evaluation must be carried out without cessation of therapy. However, due to the perplexity of the disease and its multifaceted nature, there are no specific guidelines for the timing of regimen changes or cessation of glucocorticoid therapy in children.

### Management During Adolescence

#### Patients Treated Since Childhood

Until the establishment of the normal menstrual pattern in NCCAH girls, the continuation of GCs that started during childhood is highly recommended (56). However, adolescent patients frequently do not show sufficient compliance with chronic administration of drugs and often omit doses. Additionally, during puberty, the half-life of hydrocortisone falls by 50% as a result of increased IGF-1 levels, which diminishes 11βOHSD activity, as well as due to increased cortisol clearance stemming from amplification of glomerular filtration rate (57). In the young female, 2 years post menarche and if normal ovulatory cycles have been recorded, a patient-centered approach toward the hyperandrogenic symptoms that may appear is highly recommended.

If there are severe hyperandrogenic findings, such as hirsutism, acne, and/or oligomenorrhea, continuation of treatment will be considered. It is important to remember the fragile sensibilities of a young adolescent, which will be seriously damaged by the “repellent” signs of hyperandrogenism. The combination of hyperandrogenic signs, menstrual disorders, and poor quality of life is well documented and it is particularly high in younger women. Another point to be considered is the response of adrenal reserve post ACTH stimulation. Given that peak cortisol of pubertal and adult females after stimulation is below 496 nmol/L, in cases of stress, steroid treatment should be administered (58). On the other hand, if none of the above symptoms are encountered in the young patient, GC treatment may be discontinued, after which regular follow-up is advised.

#### Females With First Manifestation During Adolescence or Hyperandrogenic Symptoms After Treatment Discontinuation

In the presence of hyperandrogenic symptoms, a patient-oriented approach is highly recommended, focusing on the main complaints of the patient. This is far better than a general universal *modus operandi*, since, with the latter approach, the young patient will be disappointed, this leading to treatment discontinuation and, as a result, to adverse health outcomes in adulthood. In cases with severe hirsutism, a course of 6 to 12 months with oral contraceptive pills (OCPs) constitutes a reasonable procedure given the beneficial effects of OCPs on SHBG liver production and the decrease of androgen release from the ovary. Clinical improvement will be expected following at least 2–3 months of OCPs initiation, while the concurrent use of a progestagen with minimal androgenic properties is highly recommended. If OCPs fail as the first line approach, antiandrogens (spironolactone, flutamide, bicalutamide, cyproterone acetate, and finasteride) may be added to the treatment. In our experience, the administration of bicalutamide has achieved significant improvement in cases of severe acne, but similar results were not obtained in cases with severe hirsutism. Cosmetic approaches such as laser application and depilatories can also be suggested for women complaining of excessive or unwanted hair growth or scalp hair loss (androgenic alopecia) (32, 34, 57).

In patients with inadequate cortisol secretion after stimulation, or if OCPs and antiandrogens cannot be tolerated, a course with GCs is highly recommended (57). Data from New et al. indicate that irregular menses and acne are reversed within 3 months after the initiation of the glucocorticoid therapy, whereas hirsutism requires nearly 30 months (59). By contrast to childhood, in adolescence, longer-acting steroids are often used and regimens of 5 mg of prednisolone or 0.25 mg of dexamethasone are recommended (53). However, in the real world clinical data have shown a variety of different regimens applied in NCCAH management (41).

The primary goal of treatment should be the patient's relief from the hyperandrogenic symptoms. Thus, among individuals with NCCAH diagnosed based solely on laboratory results, clinical features should guide management recommendations, since hormonal and molecular findings do not necessarily predict clinical severity. According to the Endocrine Society guidelines, NCCAH patients should be given the option to discontinue GC therapy when symptoms resolve (60). Of course, these patients should not be lost to follow-up, while treatment should be reinitiated in the event of recurrence of the symptoms. Further, in the case of discontinuation, patients should be informed about the possibility of infertility and should be encouraged to seek medical advice if they wish to conceive (19). Of note, the appropriate transfer of the patient from the pediatric to the adult endocrinologist should be carried out, optimally after 1 year of synchronized monitoring (53, 61).

## NCCAH and Reproduction

### Subfertility

Subfertility is commonly reported in classical forms of CAH, which is mainly due to menstrual disorders, chronic anovulation, and anatomical deformities. The birth rate has been estimated at 17% in comparison to 65% of the control population (62). Meanwhile, data regarding fertility in women with NCCAH have recently been assessed in detail and the estimated infertility incidence is 11% among NCCAH women, that is, relatively milder than in CAH, and in many cases is easily resolved. Bidet et al. evaluated fertility in 190 women suffering from NCCAH, 95 of whom wanted to become pregnant. In this population, 187 pregnancies in 85 women were reported, which resulted in 141 births in 82 individuals. It must be highlighted that 99 of the pregnancies (52.9%) occurred before the diagnosis of NCCAH, three of them with the application of ovulation induction protocols, the rest being spontaneous, while 88 pregnancies took place post NCCAH diagnosis. In the vast majority of them the pregnancy developed after the institution of therapy with hydrocortisone, whereas in 11 women it happened spontaneously (63, 64). In another report of 22 observed NCCAH women desiring pregnancy, 12 pregnancies ensued with prednisone (65).

### Miscarriages

In the cohort of Bidet et al., the miscarriage rate was 6.5 and 26.3% in patients treated with GCs and untreated patients, respectively (64). The outcome of the pregnancy may even be successful without any glucocorticoid treatment in cases where NCCAH was not yet diagnosed, as also reported in a case report by Cuhaci et al. Nevertheless, the same couple experienced two miscarriages and reported subfertility after this first pregnancy (66). As far as the possibility of preterm pregnancies is concerned, as assessed by Bidet et al., it does not differ significantly from that of the rest of the population (15).

### Fertility Planning

For those women with symptomatic hyperandrogenism or with reported infertility but who wish to conceive, GC therapy is highly recommended. During this period, the glucocorticoids used are prednisone or hydrocortisone. The most important action, because it will guide the subsequent steps, is genetic testing of the prospective parents. Females with NCCAH who desire to conceive should be aware of the risk of giving birth to an infant with the classical form of the disease. The outcome of pregnancies among women with NCCAH, and more specifically the incidence of infants born with CCAH, is estimated in recent studies to be between 1.5 and 2.5%, with NCCAH at about 15% (36, 64). Of course, this frequency depends on the reference population, a higher incidence occurring in populations with high intermarriage rates (36). The predicted chances of parents giving birth to a child with CAH is 1 in 120 for unknown paternal genotype, zero if the father has no relevant mutation, and 1 in 4 if he has a heterozygous mutation (19). As a result, genetic testing of the partners of these women is essential to assess the risk of giving birth to a child with the classical form of CAH (64, 67).

In the case that the prospective mother carries two NC mutations (Table 1), there is no absolute need for genetic testing in the future father, since, if he is heterozygous for the CYP12A2 mutations, the offspring is at risk of developing the non-classical form of the disease in the future. However, it should be noted that carrier status is 2–15% in different populations and half of these individuals carry a severe mutation. Furthermore, definitive recommendations regarding situations when genetic testing is not required are difficult given the imperfect genotype-phenotype correlation, particularly for milder mutations [81]. For example, the P30L mutation, most frequently associated with NCCAH, does also occur in patients with classical CAH [82]. A large multicentre European study recently showed that this was the case in 78% of patients [81]. In this study, the mild V218L mutation was associated with Classical rather than NCCAH in 30% of cases. Hence all patients with NCCAH should be offered genetic counseling and molecular assessment of reproductive partners.

**Table 1:** Mutations of CYP21A2 with minimal (C) or moderate (NC) residual enzyme activity (14, 68, 69).

**Classic (C) mutations**	**Non-classic (NC) mutations**
**estimated residual activity 2–10%**	**estimated residual activity 10–78%**
E380D	G424S
I235N	G375S
I172N	H62L
IVS2-13A/C>G	K121Q
G292S	P453S
Q318X	P30L
R354C	P482S
R356W	P105L
R426C	R339H
R483Q	V281L
V236E	V304M
W22X	
W406X	
Microconversions	
Deletions (30-KB DEL, 8-BP DEL)	
Large gene conversions	

By contrast, in the event that the prospective mother is a compound heterozygote with one severe (C) and one NC mutation, then genetic testing of the future father is mandatory. We should keep in mind that data originating from studies using immunoassays or the more accurate LC-MS/MS procedures, consistently highlight the inability to reliably exclude heterozygosity using basal or ACTH stimulated 17 OHP values, due to the significant overlap with the normal range. One may suggest the use of a Synacthen test, and if it is not compatible with heterozygosity (sum of basal and peak stimulated 17 OHP values < 1.5 nmol/L), then DNA testing could be avoided. However, we should be very cautious in the interpretation of this test, since it has not been validated in other studies. Hence, the use of ACTH stimulated 21-deoxycortisol either singularly or as part of a steroid ratio or steroid profile, may facilitate the biochemical identification of heterozygotes in the future, particularly as LC-MS/MS becomes more widely available [83–85]. If the prospective father carry a NC mutation, then nothing else is needed. Conversely, if he is a carrier of a Classical mutation, the question concerning prenatal treatment of the fetus will arise. All possible genotype combinations and the suggested procedures are presented in Table 2.

**Table 2:** Planning of suggested procedures during pregnancy based on prospective parents genotype.

**Genotypes**		**Procedures**
**Mother**	**Father**	
NC/NC	?	None
NC/NC	Normal	None
NC/C	?	DNA testing of future father Fetal sampling? Prenatal treatment??
NC/C	Normal	None
NC/C	NC/Normal	None
NC/C	C/Normal	Fetal sampling Prenatal treatment?

*? indicate the unknown values*.

### Prenatal Treatment of CAH

The aim of prenatal treatment of CAH is the prevention of genital virilization of the fetus, but also alleviation of the stress felt by the parents who are likely to have a child with ambiguous genitalia (70). Dexamethasone is used because of its ability to cross the placenta and because it is not deactivated from the placental 11β OH steroid dehydrogenase and binds only minimally to the mother's blood cortisol-binding globulin (CBG). Dexamethasone, by suppressing fetal ACTH secretion, decreases elevated fetal androgen production. The treatment must be discontinued in the event that karyotype or DNA analysis reveals a male or an unaffected female, respectively (71). Nevertheless, although fetal genital virilization has already started at 6–7 weeks post conception, chorionic villous biopsies for genetic diagnosis cannot be obtained before the 10th−12th week. This time interval suggests that all pregnancies at risk for virilizing CAH should be treated, even though only 1 out of 8 fetuses is affected and female (19, 34, 70).

Whether this exposure of the fetus to dexamethasone for preventive measures is medically and ethically acceptable remains controversial (70). Animal studies have demonstrated that first-trimester dexamethasone decreases birthweight, affects renal pancreatic beta cell function and brain development, and predisposes to anxiety, hypertension, and hyperglycemia in adulthood (70). Furthermore, 10–20% of pregnant women using dexamethasone treatment during pregnancy complain of weight gain, increased appetite, mood swings, insomnia, edemas, hypertension, hyperglycemia, and striae. However, New et al. found a lower than average Prader score in fetuses treated with dexamethasone prenatally, but no difference in long-term outcomes (72). Additionally, in a review by Merce Fernandez-Balsells et al., dexamethasone was shown to be associated with reduction in fetus virilization without significant maternal or fetal adverse effects (63). However, the authors of the abovementioned review as well as the consecutive guidelines of the Endocrine Society point out that because of the small sample sizes in the whole body of the literature, the subject remains uncertain and further investigation is clearly needed.

The decision about initiating treatment should be undertaken only in large centers with an experienced team and protocols approved by Institutional Review Boards and based on the family's values and preferences and with their written informed consent as a prerequisite (19, 63). Several specialists in the field indicate that a higher value should be placed on preventing unnecessary prenatal exposure of mother and fetus to dexamethasone rather than imposing the emotional toll of ambiguous genitalia on parents and patients. Most importantly, it should be clarified to parents that dexamethasone administration does not modify patient status but is directed toward reducing the need for surgery rather than preserving life or intellectual capacity. They must also know that the probability that their child will suffer from the classical form of the disease is high, despite treatment. Most crucially, meanwhile, the exposure of a young organism to a very potent GC during a particularly sensitive period of fetal programming and growth, which might well prove useless in the case of a fetus with the XY karyotype, is not at present supported by robust and unquestionable data.

Ideally, there should be an early diagnosis, this performed via a non-invasive technique and before the beginning of the genital virilization of the fetus. Working in this direction, novel studies point to the use of cell-free fetal DNA obtained from maternal plasma as a promising method that will allow the determination of fetal gender and the diagnosis of CAH at an early gestational age (<9 weeks). If this procedure is widely implemented in clinical practice, unnecessary prenatal dexamethasone treatment will be avoided (73).

### Treatment During Pregnancy

**Figure 1:**
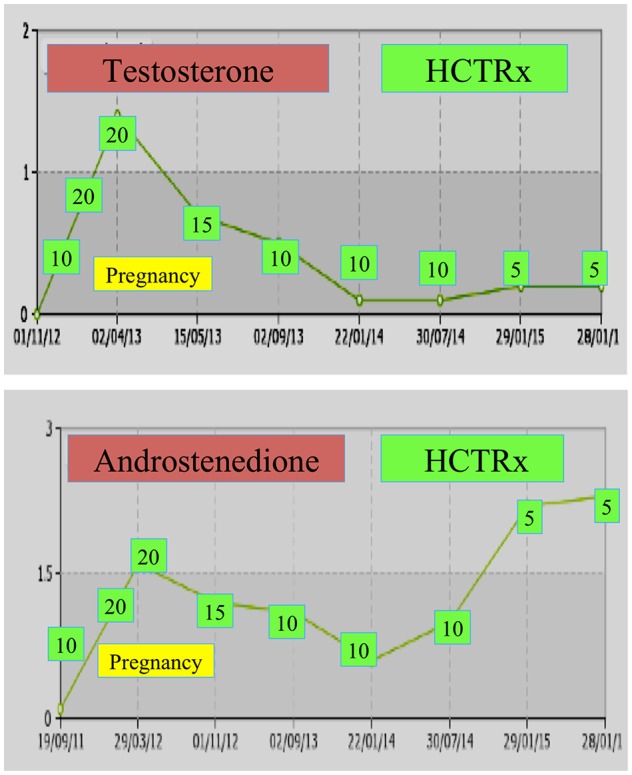
Testosterone and andostenedione levels and subsequent hydrocortisone dosing (HCTRx), from conception until 3 years post-delivery administered to a patient with NCCAH.

During pregnancy, women with NCCAH should preferably be treated with hydrocortisone, not only because pregnancy is typically accompanied by elevated stress levels, but also as a preventive measure against the abovementioned high incidence of miscarriages. This form of glucocorticoid is favored due to its metabolization by placental enzyme 11β OH steroid dehydrogenase II, which prevents glucocorticoid from having any effect on the fetus (71). At the time of conception, the hydrocortisone dose should be increased to 20–25 mg/day and dose modifications are carried out every 6–8 weeks with the aim of keeping testosterone levels at the upper normal levels of the pregnancy trimester (74). As an example, the testosterone and D4 values of a patient with NCCAH from conception up to 3 years post-delivery of two healthy twins are shown (Figure 1).

## Other Special Issues

### Stress Management in NCCAH

During major life-threatening stress, surgery, or serious illness, patients with NCCAH who are glucocorticoid-treated may require larger or more frequent doses of glucocorticoids given that their adrenal function is iatrogenically suppressed. It is therefore crucial to educate the parents of young children, as well as to re-educate patients on their transition to adult care, about stress dosing. For NCCAH patients who are not treated with GCs or in the event of discontinuation, cortisol response to ACTH should be assessed. Almost one third of NCCAH patients respond inadequately to the stimulation (15, 75). For those who respond normally to ACTH stimulation no treatment with stress doses is recommended (19). Mineralocorticoid therapy is not required in any of the cases, as these patients have normal aldosterone secretion and do not develop salt-wasting (58). Of note, in the case of hyperthyroidism or an increase in levothyroxine treatment, cortisol clearance is increased and an adrenal crisis may occur (58, 76).

### Psychobiological Aspects in NCCAH

Meyer-Bahlburg et al. have in several studies reported impaired psychological profile among patients with NCCAH due to 21OH deficiency. More specifically, the affected women had a higher masculinization/defeminization score on several measures of gender-related behavior when compared with normal control women, although markedly less so than in women with classical CAH. They also had significantly increased bisexual or homosexual orientation. Retrospective clinical-qualitative interviews with these women revealed a history of discomfort and social stress related to their pre-treatment experiences with androgen-dependent signs, such as acne, hirsutism, and conception difficulties (59, 77, 78). Similarly, Arlt et al. showed that subjective health status was significantly impaired across all eight domains of a short-term health survey, with the most prominent differences, as compared with age- and sex-matched controls, relating to the domains general health, vitality, and role limitations due to emotional problems. The Hospital Anxiety and Depression Scale (HADS) questionnaire also revealed increased anxiety scores (41). Bearing in mind these findings, psychological parameters to guide therapy should be considered in women with NCCAH and, in the context of the patient-oriented approach, a psychological diagnosis and support need to be offered.

## Conclusions

NCCAH is considered as the more frequent and milder form of CAH because of retention of 20–50% enzyme activity. Most patients may seek medical advice at any stage of their life due to symptoms related to androgen excess. These include premature pubarche, rapid growth, hirsutism, acne, menstrual irregularities, or infertility, as well as many other less prominent manifestations. Early morning baseline values of 17 OHP as a good initial screening test and further evaluation with ACTH stimulation and, in the case of borderline results, genetic testing, is recommended. As a general rule, androstenedione and not 17 OHP levels should be used during follow-up.

The treatment decision should be based on assessment of the facts and should follow an individualized approach. Treatment is not always indicated unless the patient is symptomatic, for example, children with early onset and rapid progression of pubic and body hair, rapid growth, and/or skeletal advancement, or women with oligomenorrhea, acne, hirsutism, infertility, or a combination of these and others of the abovementioned symptoms. Genetic counseling is strongly advised in NCCAH women who wish to conceive, as well as genotyping of the father. The overall management of the patient additionally includes management of the probable complications of glucocorticoid therapy or metabolism-related manifestations of the disease. Lastly, given that many therapeutic issues related to the appropriate management of these patients have not as yet been elucidated, it is very important for the attending physician to keep up to date with all developments in this field and to integrate the new data into his clinical practice. Certainly, further clinical studies in this area are essential.

To sum up, for the NCCAH woman, the ideal approach is a tailor-made one, incorporating a smooth transition of her management once she is referred from the pediatric to the adult endocrinologist, along with symptom-oriented treatment that will accompany her throughout her life. Given the multiple factors affecting the hyperandrogenic system, it is advisable to encourage patients to adopt a healthier lifestyle by improving their dietary habits, increasing exercise, and aiming at weight reduction. Furthermore, in certain cases, psychological support is often beneficial. Moreover, specialists in fields involved in the treatment of this disorder, such as dermatologists, gynecologists, and psychologists, need in-depth understanding about the management of suspicious or already diagnosed cases of NCCAH. To conclude, let us keep in mind the insightful quote of American astronomer Vera Cooper Rubin: “Science progresses best when observations force us to alter our preconceptions.”

## Author Contributions

All authors listed have made a substantial, direct and intellectual contribution to the work, and approved it for publication.

### Conflict of Interest Statement

The authors declare that the research was conducted in the absence of any commercial or financial relationships that could be construed as a potential conflict of interest.
